# Characteristics of Stress and Burnout among Lithuanian University Coaches: A Pre-Pandemic Coronavirus and Post-Pandemic Period Comparison

**DOI:** 10.3390/healthcare11142096

**Published:** 2023-07-23

**Authors:** Romualdas Malinauskas, Vilija Malinauskiene

**Affiliations:** Department of Physical and Social Education, Lithuanian Sports University, Sporto 6, LT-44221 Kaunas, Lithuania

**Keywords:** coaches, burnout elements, exhaustion, depersonalization, reduced accomplishment, perceived stress

## Abstract

(1) Background: The majority of studies analyzing associations between burnout, gender, and perceived stress have utilized a cross-sectional design. This present longitudinal study investigated associations between burnout, perceived stress, and gender compared during the pre-pandemic Coronavirus and post-pandemic period among Lithuanian university coaches. (2) Methods: 214 university coaches were randomly selected for the study. Study participants completed two measurements: one pre-pandemic and the second post-pandemic. (3) Results: Gender differences were identified on all burnout elements but not on perceived stress. Repeated measures (RM) multivariate analysis of variance (MANOVA) results did not show a significant effect for the interaction between gender and time. Results of hierarchical (stepwise) regression analyses revealed that perceived stress after the pandemic predicted burnout levels for all three burnout components (exhaustion, depersonalization, and reduced accomplishment) after the pandemic. (4) Conclusions: The current study contributes to the understanding of burnout components in relation to perceived stress through a longitudinal approach using a representative sample of Lithuanian university coaches.

## 1. Introduction

Investigating burnout in various occupations has consistently attracted much scientific consideration [1] because it is critical to identify and address its warning signs and forestall its adverse long-term health consequences. Most of the workplace reviews published so far go beyond burnout and include other work-related stress factors [1,2,3,4,5,6]. Accordingly, this study centers explicitly around these two factors—stress and burnout.

The coronavirus pandemic affected occupational health, stress, and the burnout levels of laborers both directly and indirectly: the direct effect was in dealing with risk of infection at work, and also the emerging issues connected with the post-COVID-19 condition (long COVID) that are currently being assessed [7] (p. 1); the indirect impact of the pandemic stemmed from “remote working, which has been a significant source of psychoscientific distress for laborers around the world” [7] (p. 1). Experiences of stress and burnout during the coronavirus pandemic have incited countless investigations, and a considerable number of these have detailed a high prevalence of burnout among laborers [8,9].

The majority of articles were cross-sectional, and only a few were longitudinal [9]. For example, Pijpker et al. [9] presented a study involving long-term research, consisting of two waves of data, one before and one during the pandemic [9]. The present study fills a gap in the literature; no longitudinal review has yet explored laborers’ (particularly coaches’) experienced stress and burnout before and after the pandemic (i.e., during the post-pandemic period) since only a few longitudinal reviews worldwide have examined mental health pre- and post-pandemic. These reviews revealed an increase in anxiety and depression warning signs [10] (p. 10).

Burnout is characterized in the present study as a process (or disorder) that occurs frequently among aiding professionals who work ceaselessly with individuals under circumstances in which the constant stress can be emotionally draining [11] (p. 99). The negatively experienced situations over time lead to poor mental health outcomes, such as depersonalization, emotional fatigue (exhaustion), and diminished individual achievement (loss of interest in work). Burnout is defined to be a permanent state “which occurs when a person is working prolonged and strenuously under pressure” [12] (p. 302). Most research on occupational stress and burnout has investigated them without considering a multi-faceted model. In unidimensional models, only a single aspect (fatigue) was examined. Past analysis of burnout among university coaches has utilized a unidimensional model [12]. The multidimensional model of burnout comprises three essential elements: emotional exhaustion, depersonalization, and reduced individual achievement (reduced achievement) [7,13]. Consequently, we claim that the current review fills a gap in the literature by using a multidimensional (three elements) model to analyze burnout among university coaches; this approach is similar to that of our previous work [13] on sales manager burnout.

A three-part conceptualization of burnout should be characterized and presented comprehensively. Exhaustion, with regard to burnout, refers to the profound and persistent feeling of being depleted and drained [11]. Individuals encountering fatigue might feel exhausted of energy, find it challenging to focus, frequently have reduced motivation to take part in activities, and their health status may also deteriorate [7]. Depersonalization is otherwise called emotional detachment or cynicism. People encountering depersonalization might develop a pessimistic and cynical attitude towards their partners, clients, or patients, seeing them as mere objects as opposed to people with genuine needs and emotions. As suggested by Maslach and Jackson, the third component of burnout disorder is reduced individual achievement (reduced accomplishment)—the inclination to assess oneself negatively, especially concerning one’s work with clients [11] (p. 99). Concerning burnout, people might feel an absence of efficiency, reduced confidence, and a feeling of worthlessness notwithstanding their efforts.

Past investigations have proposed that laborers are often under stress and pressure which can lead to burnout [13,14]. Within the context of this research, stress is characterized as per Hertting et al. [15], like a lasting phenomenon including the exchange of stimulation from the setting and the trainer. Perceived stress refers to “how many occasions in a particular individual’s daily routine evaluated like burdensome, uncertain, and beyond control” [16] (p. 4983). Several attributes, both unrelated and contextual, may impact coaches’ view of stressors and how they assess and respond to them [15]. In the current review, demographic (gender) and contextual viewpoints are featured as we compared perceived stress and burnout among male and female coaches during the pre-pandemic and post-pandemic periods.

Further analysis of gender distinctions is important considering that much of the research has detailed that burnout is higher in female coaches [17,18]; however, a few studies have shown it to be higher in male coaches [14], and some other investigators claim that there is no significant difference between the genders [12,19].

Past investigation before the pandemic has demonstrated that “higher perceived stress scores among athletic coaches were connected with higher emotional fatigue (exhaustion) and depersonalization and lower levels of individual achievement” [14] (p. 139). It is likely that perceived stress during the post-pandemic period will negatively affect coaches, as they may have already felt burned out while meeting their performance objectives during the pandemic, with lockdowns and ensuing return to face-to-face sport contributing to feelings of burnout [20,21]. A significant increase in the degree of stress perceived by coaches was present during the pandemic due to the experience of quarantine periods, with stress levels being fundamentally higher than those detailed in the normative data [22].

Stress and burnout among knowledge laborers [23], psychologists, and nursing students [24] in the pre- and post-pandemic periods have been analyzed in prior research. However, university coaches have not yet been comprehensively studied, and the current study, therefore, contributes to the research by investigating what changes in coach burnout levels that occurred during the pandemic could be explained by their perception of stress during the post-pandemic period.

It should be noted that university coaches were specifically vulnerable to stress during the pandemic, as its negative consequences included inadequate support for coaches, a decrease in coaches’ competence, the absence of significant interactions, insecurity about work and income, and the loss of athletes and sports programs [25]. The target of this study is university coaches as the authors have experience in this field via an observational study [12]. The investigation is conducted at the Lithuanian sports university and the sample of university coaches coincides with the mission of that particular university. It is hypothesized that female coaches were more prone to perceived stress and burnout post-pandemic, as women tend to be more sensitive to extreme situations and challenges, which is determined by their physiology and psychology [26].

The objective of the present longitudinal study is to explore associations between burnout, perceived stress, and gender compared during the pre-pandemic coronavirus and post-pandemic period among Lithuanian university coaches. This review is designed with two specific tasks. The principal task is to examine contrasts in burnout and perceived stress across gender and time (pre- and post-pandemic) in a sample of Lithuanian university coaches, with a focus on analyzing changes in three dimensions of burnout and perceived stress. In view of past studies [17,18,27], it is estimated that the elements of burnout and perceived stress were elevated in female coaches compared to their male counterparts during the post-pandemic period.

The second task of the current study aims to determine whether a significant amount of the changes in coaches’ burnout levels that occurred during the pandemic could be explained by their perception of stress. In view of past studies [14,19] we hypothesize that university coaches who perceived more significant levels of stress post-pandemic would encounter higher levels of burnout from pre- to post-pandemic.

## 2. Materials and Methods

### 2.1. Study Design

A longitudinal study design has been selected. The same cohort of university coaches completed two measurements: one during the pre-pandemic period and the second during the post-pandemic period. Measures of perceived stress and the three burnout elements were collected.

### 2.2. Study Participants and Procedure

The population of university coaches was computed utilizing the formula n = 1/(Δ^2^ + 1/N) [28], where n = the sample size, and Δ = the margin of error. In total, there were 460 coaches (this number is represented by N) coaching university athletes according to the sports federations’ lists. Required sample size was calculated in this way: n = 1/(0.0025 + 1/460) = 1/(0.0025 + 0.0022) = 1/0.00467 = 214.13; n = 214. Therefore, the required sample was at least 214 university coaches. In 2019, 240 email invitations were sent (utilizing simple probability sampling, emails were sent to every second coach on the list) and 229 coaches responded. In 2022, emails were sent to the same 229 coaches and 214 coaches returned responses (a response rate of 93.45%). Coaches’ email addresses were obtained from the university’s sports centers and the researcher emailed coaches, explaining the objectives of the current research. Email addresses were coded (to ensure anonymity) and used to match and track responses. The questionnaire was filled out by the coaches by email during the pre-pandemic period (through November and December 2019) and the post-pandemic period (through November and December 2022).

There were no contact or movement restrictions, isolations, or quarantines during the pre-pandemic period. In Lithuania, the first national quarantine was declared on 16 March 2020 and lasted until 16 June 2020. A second national quarantine was declared from the beginning of November 2020 until June 2021, during which schools, universities, and sports activities were closed. University students returned to their normal sporting activities, with some restrictions, in September 2021. The state of emergency was no longer in place from 1 May 2022. All restrictions have since been lifted. The post-pandemic period is therefore considered to start from this date.

This study received approval from the University’s Ethics Committee. Participants completed the anonymous questionnaire. Self-administered questionnaire incorporated information about coaches’ sex and age and instruments validated for usage in Lithuania to measure perceived stress and burnout elements. Data on years of education and part-time work were not collected because university coaches are graduates of the Lithuanian Sports University and part-time work is not common for university coaches in Lithuania. Data on work experience were not gathered as two-thirds of university coaches have 10 or more years of work experience and one-third have less than 10 years of work experience.

### 2.3. Perceived Stress

Perceived stress was evaluated using the Perceived Stress Scale (PSS-10), which consists of 10 items [29]. PSS-10 assessed the reactions of respondents to uncertainty, lack of control, and work pressure. These three issues form the focal elements of the experience of stress. On a five-point Likert scale, respondents reported how often they felt with a particular goal in mind. The corresponding points have been added, with higher scores demonstrating a more prominent degree of perceived stress. Tertiles were computed and used to represent ordinal scale (low/moderate/high) instead of the original continuously distributed linear score of perceived stress to allow for practical discussion of the data. Cronbach’s α was applied to assess the internal coherence of the questionnaire (0.811).

### 2.4. Burnout

Burnout elements (exhaustion, depersonalization, and reduced personal accomplishment (i.e., reduced accomplishment)) were measured using the Coach Burnout Questionnaire (CBQ), which is based on the athlete-specific ABQ [12,30] “with the wording adjusted to better suit the coaching context” [31] (p. 213). The questionnaire consists of 15 items. Respondents were instructed to assess the degree to which they encounter each item, utilizing a five-point Likert scale that ranged from 1 to 5. For each burnout element, a mean score (burnout element indicator) has been calculated. A higher score indicates greater burnout. Tertiles were computed and used to represent ordinal scale (low/moderate/high) instead of the original continuously distributed linear score to allow for practical discussion of the data. Cronbach’s α for the burnout elements were, respectively, 0.759, 0.712, and 0.770.

### 2.5. Statistical Analysis

The factors were checked for normality (skewness and kurtosis coefficients were determined; these coefficients, ranging between −1 and +1, are considered to indicate that data are regularly published). Two-tailed Pearson correlations were determined for the review factors. A 2 (gender: male; female) × 2 (time: before the pandemic, T1; after the pandemic, T2) repeated measures (RM) multivariate analysis of variance (MANOVA) was applied to explore how time and sex impacted burnout and perceived stress among university coaches. Hierarchical (stepwise) linear regression analysis was used to uncover whether many of the changes in coaches’ burnout levels that occurred across the pandemic could be explained by their perception of stress after the pandemic (T2). The initial step of regression included the burnout element at T1 as a predictor of the burnout element at T2. This meant that the dependent variable (burnout element) actual score at T2 controlled for the baseline score at T1 in the linear regression. The second step included the burnout element at T1 and perceived stress at T2. The use of stepwise linear regression analysis for two waves of data analysis is still an area of debate among researchers. Although some researchers advise not to use stepwise regression methods to analyze two-wave data, some other researchers have used stepwise regression analysis for two waves of data. For instance, stepwise regression analysis was used for the investigation of a two-wave Scottish cohort study during the pandemic [32]. However, stepwise regression could be less effective when a larger number of potential explanatory variables is used [33]. Nevertheless, this is not the case in this study. Stepwise regression analysis can also be used in the current study instead of mediational analysis because the present study aims to determine what amount of the changes in university coaches’ burnout levels that occurred during the pandemic, could be explained by their perception of stress during the post-pandemic period (T2). The mediation model may be more appropriate to explore the mediating effect of perceived stress on the relationship between burnout at T1 and at T2, controlling for perceived stress at T1, where the aim will be to assess the overall effect of perceived stress on burnout levels (but not the effect of perceived stress at T2).

All assumptions for the application of MANOVA have been met. Impact sizes for F-statistics were communicated as η_p_^2^. SPSS 28.0 package was used for the statistical analyses.

## 3. Results

The current study included 138 male and 76 female university coaches, with an average age of 36.84 ± 9.92. The analysis of the distribution of participants according to the levels of the indicators (low, medium, high) reveals that the sample of 214 university coaches reported high levels of burnout components (exhaustion, 44.3%; depersonalization, 41.5%, reduced accomplishment 37.9%) and perceived stress (45.8%) at baseline. For the second measurement, coaches also rated the level of burnout components as high: exhaustion, 47.7%; depersonalization, 45.3%, reduced accomplishment 44.9%. The proportion of participants meeting the threshold for high levels of perceived stress increased to 48.1% at follow-up.

Pearson correlations were calculated between study variables (Table 1). The correlation matrix of the dependent variables confirmed the absence of multicollinearity, which is consistent with one of the assumptions of MANOVA (“MANOVA is deemed inefficient in cases where the dependent variables exhibit either extremely high positive correlations or lack of correlation”) [34] (p. 223). Correlations are presented in Table 2: below the diagonal are for time before the pandemic (T1) and above the diagonal are for time after the pandemic (T2). The dependent variables are not very highly correlated in a positive direction.

Exhaustion was strongly correlated with every variable in the study, and the strongest correlation was found with reduced accomplishment (0.432, *p* < 0.01) for T2. Devaluation and reduced accomplishment also positively correlated with all study variables (*p* < 0.01) during T2 and (*p* < 0.05) during T1. The lowest correlations were found between perceived stress and two burnout elements (exhaustion (0.142, *p* < 0.05) and reduced accomplishment (0.136, *p* < 0.05)) at T1.

The assumptions of sphericity and homogeneity of variance for RM MANOVA were checked using Mauchly’s test of sphericity and Levene’s test, and found to be satisfactory. Overall, the RM MANOVA yielded a small main effect for gender (*F*(4, 209) = 3.17, *p* = 0.015; Wilk’s λ = 0.94; η_p_^2^ = 0.06). A multivariate test indicated that time did affect burnout elements and self-perceived stress (*F*(4, 209) = 7.58, *p* = 0.0001, Wilk’s λ = 0.87; effect was medium η_p_^2^ = 0.13). Overall, the RM MANOVA results did not show a significant effect for the gender by time interaction (*F* (4, 209) = 0.18 *p* = 0.949; Wilk’s λ = 1.00; η_p_^2^ = 0.003).

Univariate *F*-tests showed gender differences on all burnout elements (Table 2) but not on perceived stress (*p* = 0.076). Simple effects analysis showed that female university coaches reported higher exhaustion (*D* = 0.19, *p* = 0.038), depersonalization (*D* = 0.13, *p* = 0.050), and reduced accomplishment (*D* = 0.14, *p* = 0.029) than males at T2. Univariate analysis revealed a small effect (η_p_^2^ = 0.05) of time on fatigue (*D* = 0.17, *p* = 0.0001) and a medium effect (η_p_^2^ = 0.10) of time on perceived stress (*D* = 1.74, *p* = 0.0001).

Pre-pandemic burnout elements and post-pandemic perceived stress measures were considered as the predictor variables (Table 3). We did not include gender in the regression models because we did not find a significant effect for gender by time interaction. For all three hierarchical regression analyses, the burnout elements at T1 were used as a predictor of the burnout elements at T2 and were included in the first stage of the stepwise linear regression analysis, and the measures of coaches who perceived stress at T2 were added at the second stage. A variance inflation factor (VIF), used to measure the amount of multicollinearity in regression analyses, was checked. All three hierarchical regression analyses had a VIF value of 1 indicating the absence of multicollinearity.

For the main regression analysis, in which exhaustion at T2 was the dependent variable, the addition of exhaustion at T1 as the indicator at Stage 1 (i.e., Step) uncovered a critical predictive impact, *F*(1, 212) = 61.85, *p* < 0.001; R^2^ = 0.23. At Stage 2, the addition of perceived stress at T2 indicated a critical rise in estimated variance (R^2^ change = 0.02, *F*(1, 211) = 6.88, *p* = 0.009), recommending that perceived stress at T2 contributed substantial prognostic value for exhaustion at T2. A simple regression analysis model explained 24% of the variance (adjusted R^2^ = 0.24) in university coaches’ exhaustion scores estimated post-pandemic. “This index of impact size is viewed as medium” [35] (p. 413) considering that for R^2^ the huge value is 0.26 [35] (p. 413).

The initial step of the second regression included devaluation at T1 as a predictor of devaluation at T2. Devaluation at T1 as a predictor yielded a massive impact (R^2^ = 0.19, *F*(1, 212) = 48.95, *p* < 0.001). At Stage 2, the addition of perceived stress at T2 indicated a huge increase in estimated variance (R^2^ change = 0.13, *F*(1, 211) = 38.88, *p* < 0.001), recommending that perceived stress at T2 contributed substantial prognostic value for devaluation at T2. A simple regression analysis model explained 31% of the variance (adjusted R^2^ = 0.31) in university coaches’ devaluation scores estimated post-pandemic, indicating a huge impact size [35].

For the third regression investigation with reduced accomplishment at T2 as the dependent variable, the addition of reduced accomplishment at T1 as an indicator at Stage 1 uncovered a huge impact (R^2^ = 0.13, *F*(1, 212) = 32.86, *p* < 0.001). At Stage 2, the addition of perceived stress at T2 showed a huge addition in estimated variance (R^2^ change = 0.06, *F*(1, 211) = 16.60, *p* < 0.001, recommending that perceived stress at T2 contributed substantial prognostic value for reduced accomplishment at T2. A simple regression analysis model explained 19% of the variance (adjusted R^2^ = 0.19) in university coaches’ reduced accomplishment scores estimated post-pandemic, indicating a medium impact size [35].

## 4. Discussion

This longitudinal study’s primary aim was to explore associations between burnout, perceived stress, and gender compared during the pre-pandemic Coronavirus and post-pandemic period among Lithuanian university coaches. The research hypothesis, i.e., that burnout and perceived stress would be higher in female coaches than in males post-pandemic, was partially confirmed. Analysis of the data showed that female university coaches scored higher on all elements of burnout (exhaustion, depersonalization, and reduced accomplishment) than males at the post-pandemic assessment (with small effect sizes). Although gender differences were not identified in perceived stress, notably, findings from the current study are consistent with the results of another study conducted with athletic coaches during the pandemic period [19]. Data by Singe et al. [19] revealed that female coaches displayed greater levels of burnout than male coaches (small effect size; *d* = 0.29). This discovery is bolstered not just by our study’s outcomes but also in other research [33,36], which revealed that female coaches had higher levels of burnout across all burnout dimensions. Reasonably, “burnout differences between men and women are not isolated to athletic coaches, as women in various health care fields noted greater levels of burnout than men” [19] (p. 1097).

Stress related to COVID-19 “may worsen pre-existing mental health and contribute to the onset of new traumatic conditions related to stress” [37] (p. 331) for some and particularly for coaches. In the present review, distinctions in gender were not identified on perceived stress post-pandemic, a finding that corresponds with other analyses. For example, a recent study by Hertting et al. [15] found measurably non-significant differences between perceived stress among male and female soccer coaches (with a very small effect size; *d* = 0.13). Furthermore, another study, which investigated athletic coaches during the pandemic, delineated comparative degrees of perceived stress among coaches, and female coaches were not found to have statistically different degrees of perceived stress [38].

Only a limited number of studies have provided pre- and post-pandemic comparisons of coaches’ perceived stress; therefore, analyzing studies from different sports professionals or other professions would be valuable. Notably, correlations of other comparative studies have been made with data during the pandemic, but not after it [39]. Antoniadou [39] found that during the pandemic, individual fatigue was impacted by gender; however, no distinctions in stress indicators were highlighted. Di Fronso et al. [40] compared the perceived stress of Italian athletes before and during the pandemic and found that female athletes had higher perceived stress scores than male athletes during the pandemic, comparing to those before pandemic (effect size determined utilizing partial eta square was small η_p_^2^ = 0.05). Another review [41], which evaluated contrasts between the perceived stress of various sociodemographic groups of students prior to and during the pandemic, showed that female students reported more stress (with a small effect size). Taken together, these insights suggest that gender differences in perceived stress were possibly found while comparing stress data before and during the time of the pandemic (and not post-pandemic).

The current study confirmed the second hypothesis that university coaches who had higher levels of perceived stress post-pandemic would experience higher levels of burnout from pre- to post-pandemic. The results of hierarchical (stepwise) regression analyses revealed that perceived stress after the pandemic (T2) contributed significantly to the prediction of university coaches’ burnout levels for all three burnout elements (exhaustion, depersonalization, and reduced accomplishment) post-pandemic and effect size varied from medium, for exhaustion and reduced accomplishment, to large, for depersonalization.

These findings are consistent with the theoretical points, which “centers on burnout, arising from prolonged workplace stress” [42] (p. 1) and with empirical evidence in the study by DeFreese et al. [43], which found that perceived stress, along with social support, drive all burnout elements (exhaustion, depersonalization, and reduced accomplishment). This suggests that stress perceptions contribute to the experience of burnout among coaches. Effect sizes were large for exhaustion (R^2^ = 0.49) and depersonalization (R^2^ = 0.29) and small for reduced accomplishment (R^2^ = 0.10) [43].

The field of perceived stress and burnout associated with COVID-19 should incorporate new investigations that will emerge now and well into the future. The findings of the present study and the study by DeFreese et al. [43] have confirmed the need to further investigate perceived stress as a predictor of all three burnout dimensions (exhaustion, depersonalization, and reduced accomplishment). Future burnout studies should take these recommendations into account.

### Strengths and Limitations

One of the current study’s strengths is the longitudinal study design; the same cohort of university coaches completed measures at two time points: prior to the pandemic and after the pandemic. Another strength of the current study is that it sampled for a 95% confidence level, which ensured the reliability and representativeness of the data in the Lithuanian context.

This study only used survey methods and only university coaches were examined, both of which are limitations. Further investigation should examine coaches working with young athletes and high-performance athletes. Additional research is necessary to comprehend the prolonged exposure of the pandemic on university coaches.

The use of stepwise linear regression analysis for two-wave data instead of mediation analysis can also be interpreted as a limitation. Some researchers warn against the use of stepwise regression methods due to their potential to increase sampling error, leading to overfitting and poor out-of-sample prediction accuracy [33]. To avoid these issues, future researchers should consider opting for mediation analysis rather than stepwise regression analysis for two-wave data.

The study is limited with regard to study population demographics data. Only age, gender, occupation (per design), and the questionnaires were collected as data. As it is known that several other factors are associated with stress and burnout (e.g., social support, marital status, years of education, and work experience [44]), further research should collect and control for more demographic data that could have implications for burnout and stress.

## 5. Conclusions

The current study contributed to the investigation of burnout components (exhaustion, depersonalization, and reduced accomplishment) and perceived stress using longitudinal approach in a representative sample of Lithuanian university coaches. Gender differences were identified on all three burnout components but not on perceived stress. The results of the current study indicate that exhaustion and perceived stress levels in university coaches change from pre-pandemic COVID-19 to post-pandemic period. A significant effect for the gender by time interaction was not found. All three burnout components at Time before the pandemic correspondingly were predictors of burnout components at Time after the pandemic. The results of regression analyses revealed that perceived stress at Time after the pandemic made a considerable contribution to predict of university coaches’ burnout for all burnout components at Time after the pandemic. Substantially, the level of burnout components at baseline and follow-up practically means that university coaches needed more attention from policymakers and practitioners. In line with a result of the current study, we emphasize the need for practitioners to develop and implement stress coping skills training programs, and further promote resilience of university coaches in coping with stress and burnout.

These conclusions may be useful to understand that interventions to reduce burnout should focus on organizations rather than individuals. The main causes of burnout tend to be organizational factors, such as stressful situations at work or work overload. Organizations should regularly assess the level of stress and burnout experienced by their employees.

## Figures and Tables

**Table 1:** Correlations of study variables (burnout (CBQ) elements and perceived stress).

	1	2	3	4
1. Exhaustion	1	0.378 **	0.432 **	0.164 *
2. Devaluation	0.414 **	1	0.380 **	0.427 **
3. Reduced accomplishment	0.336 **	0.221 **	1	269 **
4. Perceived stress	0.142 *	0.163 **	0.136 *	1

Correlations below the diagonal are for time before the pandemic (T1). Correlations above the diagonal are for time after the pandemic (T2). N = 214. * *p* < 0.05; ** *p* < 0.01. CBQ—Coach Burnout Questionnaire.

**Table 2:** Means (*M*) and standard deviations (*SD*) for burnout (CBQ) elements and perceived stress across gender groups and time.

	T1		T2		Univariate Tests of RM MANOVA					
	Males	Females	Males	Females	Gender		Time		Gender × Time	
	(*M* ± *SD*)	(*M* ± *SD*)	(*M* ± *SD*)	(*M* ± *SD*)	*F* (*df* = 1,212)	η_p_^2^	*F* (*df* = 1,212)	η_p_^2^	*F (df =* 1,212)	η_p_^2^
Exhaustion	1.96 ± 0.61	2.21 ± 0.59	2.14 ± 0.64	2.33 ± 0.62	8.47 **	0.04	12.05 **	0.05	0.44	0.00
Devaluation	1.99 ± 0.44	2.12 ± 0.46	2.03 ± 0.46	2.16 ± 0.48	5.40 *	0.03	1.11	0.01	0.01	0.00
Reduced accomplishment	2.53 ± 0.48	2.68 ± 0.52	2.61 ± 0.47	2.75 ± 0.38	6.95 **	0.03	3.69	0.02	0.03	0.00
Perceived stress	22.69 ± 3.19	21.53 ± 4.22	22.55 ± 3.32	23.04 ± 3.90	3.19	0.02	23.56 **	0.10	0.25	0.00

* *p* < 0.05; ** *p* < 0.01. T1: pre- pandemic, and T2: post-pandemic. CBQ—Coach Burnout Questionnaire.

**Table 3:** Hierarchical (stepwise) linear regression analyses results for burnout (CBQ) elements.

Step	Dependent Variable	Predictor Variable(s) Entered	R^2^	R^2^ Change	*F*-Change	df1	df2	Beta
1	Exhaustion (T2)	Exhaustion (T1)	0.23	0.22	61.85 **	1	212	0.48 **
2		Exhaustion (T1)	0.27	0.02	06.88 **	1	211	0.47 **
		Perceived stress (T2)						0.16 **
1	Devaluation (T2)	Devaluation (T1)	0.19	0.19	48.95 **			0.43 **
2		Devaluation (T1)	0.31	0.13	38.88	1	212	0.37 **
		Perceived stress (T2)				1	211	0.36 **
1	Reduced accomplishment (T2)	Reduced accomplishment (T1)	0.13	0.13	32.86 **			0.37 **
2		Reduced accomplishment (T1)	0.20	0.06	16.60 **			0.35 **
		Perceived Stress (T2)				1	212	0.25 **

T1: pre-pandemic. T2: post-pandemic. ** *p* < 0.01.

## Data Availability

The datasets collected and analyzed during the current study are available from the corresponding author on reasonable request.
